# Delay-Sensitive Multi-Sensor Routing Scheduling Method for Underground IoT in Mines

**DOI:** 10.3390/s25020369

**Published:** 2025-01-10

**Authors:** Yinghui Zhang, Mingli Liu, Aiping Tan

**Affiliations:** 1 School of Computing and Software, Chengdu Neusoft University, Chengdu 611844, China; zyh@nsu.edu.cn; 2 School of Information Management and Business Administration, Chengdu Neusoft University, Chengdu 611844, China; 3 School of Cyber Science and Engineering, Liaoning University, Shenyang 110036, China; aipingtan@lnu.edu.cn

**Keywords:** real-time schedule, delay-sensitive, underground mine, Internet of Things, multi-hop routing scheduling

## Abstract

Recently, there has been a growing interest in underground construction safety, during activities such as subway construction, underground mining, and tunnel excavation. While Internet of Things (IoT) sensors help to monitor these conditions, large-scale deployment is limited by high power needs and complex tunnel layouts, making real-time response a critical challenge. A delay-sensitive multi-sensor multi-base-station routing scheduling method is proposed for the IoT in underground mining. First, a mixed network topology of wireless and wired networks is formed based on the irregular distribution characteristics of multiple tunnels in the mine construction environment. Based on this topology, a multi-sensor and multi-base-station real-time routing scheduling problem is proposed, proving that the problem is NP-hard. Secondly, the corresponding solving algorithms are designed based on the greedy strategy and the heuristic strategy. Finally, an experimental platform is built, and the performance of the proposed algorithm is compared and analyzed.

## 1. Introduction

Underground mining is an industry with a high accident rate that presents significant risks to both workers and operations. The persistent challenges of preventing mining accidents, detecting them in real time, and mitigating their consequences has received increasing attention from both researchers and industry professionals [1]. Recently, advances in mining safety have focused on the integration of technology, particularly in predicting and preventing accidents before they occur. The early detection of hazards and minimizing the impact of incidents remain critical and complex areas of study [2]. A key aspect of these advances is the timely and accurate transmission of information within mines, making the Internet of Things (IoT) an essential tool in enhancing operational safety.

In the context of mining, IoT sensor networks are indispensable for monitoring and managing critical data, including the status of equipment, personnel locations, and environmental conditions. These networks not only improve the efficiency of mine operations, but also significantly improve safety by detecting potential hazards before they escalate. IoT devices provide real-time data that enable predictive maintenance and early anomaly detection, thus preventing accidents. In addition, in the event of an accident, these sensor networks accelerate the transmission of key information, aiding in a faster emergency response and improving the overall effectiveness of rescue operations. By relaying detailed event data, the impact on post-incident construction and recovery efforts is minimized.

However, despite the potential of IoT in mining, wireless communication in such environments faces two primary challenges. First, data transmission delays occur because data packets often pass through multiple network nodes, reducing the timeliness of critical information. Second, data collisions caused by simultaneous transmissions from multiple sensors lead to packet loss, which compromises the reliability of the communication system. These challenges highlight the need for robust and efficient wireless transmission methods tailored to the demanding conditions of underground mines.

To address these issues, various researchers have investigated routing and scheduling algorithms designed for use in underground mining environments. Chehri [3] and Wu [4] have explored ways to optimize power consumption and extend the network lifetime of Wireless Sensor Networks (WSNs) by strategically placing sensors and applying quality-of-service strategies. Their research has focused on improving the reliability and scalability of the networks through load-balancing techniques. In particular, Chehri et al. [3] accounted for sensor mobility and channel characteristics, offering a comprehensive analysis of real-world applications.

In addition, Chen et al. [5] proposed a hierarchical communication structure, which organizes the network into separate layers of the protocol stack for communication and information transmission. This approach improves scalability and improves network robustness by optimizing resource utilization between routing nodes. Zhang et al. [6], on the other hand, introduced a delay-sensitive routing and scheduling method based on Ant Colony Optimization (ACO). Through simulations and performance comparisons, Zhang’s method demonstrated superior performance in minimizing delay and jitter under specific mining conditions.

Despite the progress made, several challenges remain unresolved in current research. One major limitation is the insufficient consideration of transmission delay, as most studies emphasize energy efficiency and packet loss without addressing the varying transmission deadlines of different sensors. The lack of comprehensive delay analysis presents a gap in existing solutions, especially in time-sensitive applications.

This paper aims to address these gaps by presenting the following novel contributions:

- Based on the unique characteristics of underground construction environments, a hybrid network topology that integrates wireless sensor collection with wired transmission is proposed. A real-time routing and scheduling method is proposed, taking into account the transmission deadlines of individual sensors. This problem is proven to be NP-hard;
- We introduce algorithms based on both greedy and heuristic strategies and improve traditional shortest path algorithms to better suit the complex conditions in mining environments. Comparative analysis in various experimental setups demonstrates that the proposed algorithms significantly reduce transmission delays across a range of application scenarios.

The remainder of this paper is structured as follows. Section 2 reviews related work in the field of WSNs in underground mining. Section 3 presents the system model and formalizes the problem definition. Section 4 describes the design of the proposed algorithms. Section 5 provides a detailed experimental analysis and a comparison of the algorithms’ performance. Finally, Section 6 concludes the paper, summarizing the key findings and outlining potential directions for future research.

## 2. Related Work

Currently, there is a substantial amount of research on wireless sensor routing and scheduling, encompassing various applications in different scenarios. For example, WSNs are deployed in underwater environments [7], mobile scenarios [8], and acoustic sensor networks [9]. These scenarios present unique challenges, requiring tailored approaches for optimizing routing and scheduling. Broadly, the routing and scheduling algorithms for WSNs can be classified into the following categories.

### 2.1. Energy-Based Wireless Sensor Routing Algorithms

Energy consumption is a critical factor in the design and operation of WSNs, especially when sensor nodes are deployed in remote or inaccessible areas. Numerous studies have developed energy-efficient routing algorithms to reduce power consumption while maintaining network performance, each offering unique strategies to tackle this challenge.

One approach utilizes optimization techniques, such as the genetic algorithm presented in [10], which targets key metrics like network lifetime, throughput, delay, and reliability. This algorithm identifies optimal routing paths in a graph structure, effectively balancing energy consumption with transmission performance. Similarly, regional management schemes, such as the divide-and-conquer strategy proposed in [11], focus on subdividing the network into smaller regions to manage energy consumption locally. By distributing energy demands across these regions, this method extends the operational life of the network and adapts well to large-scale WSNs with varying node energy levels.

Machine learning has also been applied to enhance energy efficiency, with methods like the Q-learning-based, data aggregation-aware algorithm in [12]. This approach leverages the aggregation degree of neighboring nodes to reduce redundant transmissions, thereby conserving energy and dynamically adjusting routing paths for optimal network longevity. The combination of learning-based and optimization strategies demonstrates that both dynamic adaptation and regional energy management are effective in reducing power usage across WSNs.

However, these strategies often assume relatively stable topologies and do not fully account for the complexities of challenging environments, such as underground mines. In such settings, factors like sensor mobility, dynamic topology changes, and harsh conditions pose additional challenges that require both energy efficiency and robustness for sustained operation. While these existing methods provide foundational techniques, they fall short of addressing the unique demands of underground mining applications, where continuous operation under extreme conditions is essential.

### 2.2. Delay-Based Wireless Sensor Routing Algorithms

Another significant research focus in WSNs involves minimizing transmission delays, a crucial requirement in many IoT applications such as real-time monitoring and emergency response systems. Low-latency communication is particularly essential in environments like underground mines, where timely data transmission directly impacts both safety and operational efficiency.

Various delay-optimized routing protocols have been proposed to address these challenges. For instance, the delay-sensitive routing algorithm in [13] employs relay nodes strategically to minimize transmission delays and extend network lifetime. Using game-theoretical principles to optimize relay node selection, this method reduces the number of hops and routing overhead, thereby enhancing throughput and achieving low-latency performance. This approach is particularly relevant in settings like mines, where even minor delays in data transmission could lead to safety hazards.

Location-aware and opportunistic routing strategies also offer significant benefits for delay reduction in dynamic networks. As seen in [7], a location-based opportunistic routing protocol leverages node positions to minimize transmission delays in underwater sensor networks. By ensuring that data packets take the shortest available path, the protocol reduces both delay and energy consumption while dynamically adapting to changing network conditions. This adaptability makes it suitable for environments requiring high responsiveness and energy efficiency, as is often the case in WSNs deployed in underground or challenging terrains.

Similarly, cluster-based methods have proven effective in delay and energy management. The cluster-based data aggregation scheme in [14] constructs an aggregation tree and uses time-slot scheduling to mitigate retransmissions and waiting times. This structure enables faster transmission, reduced congestion, and lower overhead, making it particularly advantageous in networks with high sensor density and simultaneous data transmissions.

Analyzing these approaches reveals that optimizing the Medium Access Control (MAC) layer is a common strategy for addressing delay issues. Techniques such as Time-Division Multiple Access (TDMA) are frequently employed to manage traffic flow in a controlled manner, allowing for predictable data scheduling and minimizing transmission delays. However, TDMA requires some advance knowledge of data flow characteristics, such as flow count, deadlines, and data sizes, which may not always be feasible in real-world scenarios with dynamic data requirements.

On the other hand, contention-based methods, like Carrier-Sense Multiple Access (CSMA), are also widely used for data access and routing in WSNs. While CSMA is flexible and effective in managing unscheduled traffic, it may introduce packet collisions and delays, especially in high-density sensor environments such as underground IoT networks. In such cases, where reliable and low-latency communication is essential, contention-based approaches often struggle to meet stringent real-time requirements due to the increased likelihood of transmission conflicts and congestion.

The methods discussed highlight a range of strategies for reducing transmission delays across different WSN applications. However, many of these approaches require specific network configurations or assume relatively stable conditions, which limits their effectiveness in complex environments like underground mines. This study builds on these foundations by adapting delay-optimized routing strategies to handle the unique challenges of underground IoT networks, where both delay minimization and robust performance are critical.

### 2.3. Age of Information in IoT-Based Real-Time Monitoring

Age of Information (AoI) is an increasingly important metric in IoT applications, particularly in scenarios that rely on timely, accurate data updates to support real-time decision-making and rapid response, such as the monitoring systems used in underground mines [15]. Unlike traditional delay metrics, AoI focuses on the “freshness” or “age” of the information that reaches the decision-making systems, measuring how current the data are at any given time. This is especially critical in high-stakes environments like mining, where outdated information on toxic gas levels, structural integrity, or water infiltration could lead to hazardous incidents and pose severe risks to personnel safety. Ensuring that the information available to the system remains as up-to-date as possible can make a significant difference in the ability to anticipate and respond to threats before they escalate.

Recent research on AoI in IoT networks has concentrated on methods to optimize data update frequencies and reduce latency, with the goal of maintaining data relevance over time [16,17]. One prominent approach is AoI-minimal clustering, which groups sensor nodes strategically to minimize the age of data received across various clusters. By organizing sensors into well-planned clusters, it is possible to streamline data flow from each cluster, reducing information latency and enhancing the efficiency of data retrieval. This clustering approach has shown promise in large-scale IoT networks by managing the trade-offs between data update rates and network resource constraints, though it typically requires adaptation to work effectively in underground mine environments with their unique layouts and connectivity challenges.

In addition to clustering methods, other studies have explored AoI-oriented optimization strategies in different types of IoT and wireless networks. For example, techniques such as transmission and trajectory co-design for Unmanned Aerial Vehicle (UAV)-assisted Wireless Powered Communication Networks (WPCNs) [18] offer valuable insights into optimizing both the physical paths of data transmission and the frequency of updates in settings where nodes are mobile or the environment is highly dynamic. Although these strategies were originally designed for UAV applications, they present opportunities for adaptation in fixed underground sensor networks. Specifically, by applying similar principles to optimize transmission routes and adjust update intervals, it becomes possible to achieve high efficiency in data updates in the context of a mixed wired–wireless system, as is commonly found in mine networks.

For underground mines, where sensor nodes may have limited energy resources and a challenging deployment layout, adapting these AoI-related techniques can be transformative. By strategically clustering sensor nodes and carefully planning transmission paths, the freshness of critical safety information can be maintained with minimal delay, thereby improving the real-time responsiveness of the IoT network in a power-efficient manner. This paper builds on the principles of AoI minimization to address the unique demands of underground mine monitoring, providing a solution that enhances both the timeliness and reliability of data collection for improved mine safety management.

In addition, there are some studies focused on real-time transmission and scheduling in wireless networks [19,20,21,22]. However, the application of these results in underground mines faces several challenges. In conclusion, while both energy-based and delay-based wireless sensor routing algorithms have seen significant advancements, there are still considerable challenges in applying these methods to more complex and dynamic environments like underground mines. In such settings, the network topology is not fixed, and sensors often face harsh physical conditions, making it difficult to maintain efficient, reliable communication. There is a need for further research to develop more adaptable algorithms that can address both energy and delay challenges while ensuring robust, real-time data transmission in underground IoT applications.

## 3. System Modeling

### 3.1. Topology Structure of Underground IoT in Mines

**Figure 1:**
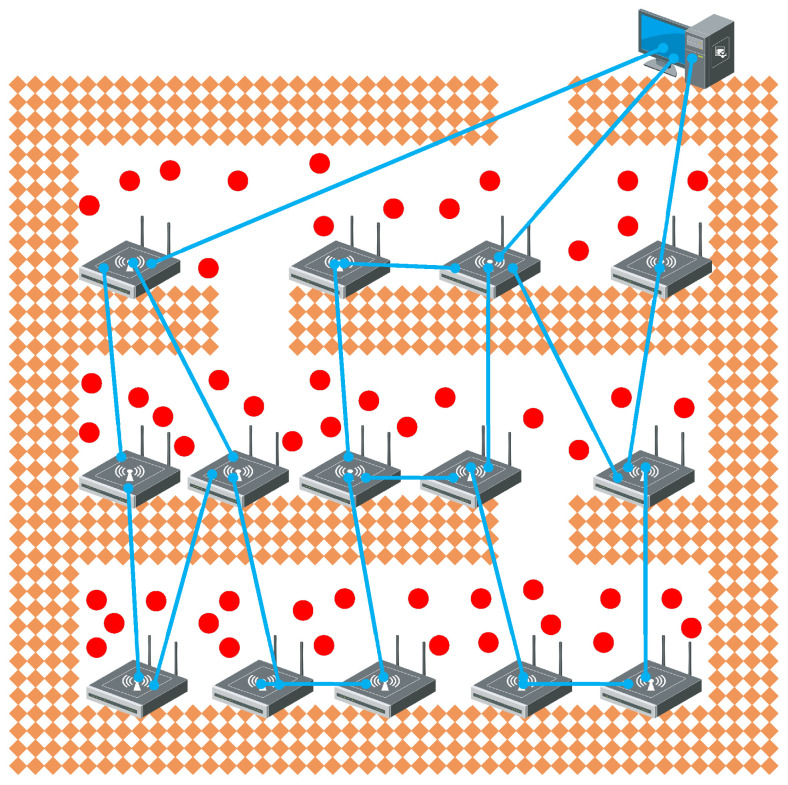
The hardware deployment diagram of the IoT under the mine.

IoT devices are deployed along irregular tunnels in some complex construction environments with multiple tunnels, such as metal mining sites. As shown in Figure 1, in the process of gold mining, tunnels need to be excavated according to the distribution of veins, so the deployment of IoT devices will present a mesh topology along different tunnels.

In Figure 1, the base stations are connected to each other via wired connections, while the sensor still sends data wirelessly. The base station and the sensor form a star topology, and the base station and the base station present a multi-hop network topology according to the actual situation of the tunnel. In this topology, both the reliability of wireless transmission and the path of multi-hop data transmission between base stations need to be considered to ensure that data are sent to the cloud between deadlines.

**Figure 2:**
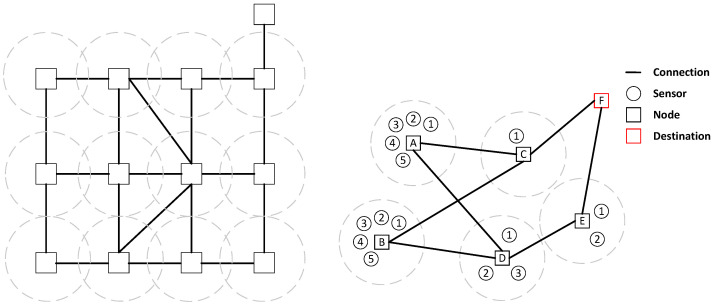
Hybrid topological structure of mesh and star.

Based on the IoT application scenario in underground construction, Figure 2 is a multi-sensor IoT application topology. In this topology, node F is the endpoint of the cloud device. The four base stations A,B,C, and *D* form a mesh topology through a cable. The sensor (1, 2, 3, …) sends wireless data to the base station, and the base station needs to send data to the endpoint *F* through the multi-hop network topology. Assume that the No. 1 sensor of the base station *A* sends data to *A*, and *A* can send data to *F* through multiple path selections. When the network does not consider other node data, the transmission problem is the shortest-path problem, which the shortest-path algorithm can solve. However, when other nodes in the network also need to send data, considering the delay caused by node caching, queuing, etc., how to send all data to focus *F* in the shortest time is a problem that needs to be studied such as in this paper.

As shown in Figure 2, the next hop of the packet arriving from the base station *A* can select the base station *C* or the base station *D*. After selecting the base station *C*, the direct path to the destination base station is the shortest path. If base station *A* has only one data packet, and there is no other data packet except base station *A*, this method can obtain the minimum delay. However, in practice, if all data packets choose the shortest path to reach the destination base station, such as base station *A* and base station *B*, all data packets of the base station are through the base station *C*, which will lead to congestion at the base station *C*. Compared with the case where each data packet randomly selects the path based on not repeating the path, the delay will be higher. If all data packets are routed to the base station with the smallest load at the next hop, there will likely be an unnecessary detour. Assume that the load of the base station *D* is slightly smaller than that of the base station *C*; a single data packet in the B base station will select the base station *D*. Since the load of the next hop of base station *D* and the number of hops required cannot be known directly, the next-hop base station *E* of base station *D* may produce a higher delay after the base station *D* is selected by detour. As a result, the delay of a single packet is not as good as the shortest path. In scheduling, data packets will interact with each other. Using a fixed decision method to optimize the individual scheduling data packets in the front is likely to lead to a significant deterioration of the scheduling results of the data packets in the back of the scheduling problem. For example, it is assumed that the base station *A* has two packets with an arrival time of 0 and a deadline of 3. The base station *E* has one packet with an arrival time of 2 and a deadline of 1. If the two packets of the base station *A* evenly select *C* and *D* as the next hop, at the time of 2, one of the packets reaches *E*, but the one packet that the base station *E* reaches cannot reach the target base station on time. If both packets of base station *A* pass through base station *C*, all packets can arrive before the deadline. Therefore, scheduling from a global perspective is the main starting point of this paper.

### 3.2. Multi-Sensor Real-Time Routing Scheduling Problem

Assume that there are a total of *I* base stations; for any 0≤i≤I−1, nbr(i) denotes the set of all nodes connected to node *i*. Obviously, for $0\leq i^{\prime }\leq I-1$, if $i\in nbr(i^{\prime })$, then $i^{\prime }\in nbr\left(i\right)$. Let *e* be the set of export nodes. Suppose that there are a total of *J* data packets in the periodic time of *T* time slots (assuming that all nodes can transmit a data packet within a time slot). For 0≤j≤J−1, use $B_{j}$ to represent the base station to which the data packet *j* belongs (issued by which base station), the arrival time of the data packet *j* is $A_{j}$ (the arrival time of the data packet *j* to the base station), the deadline is $D_{j}$, and the absolute deadline is $A_{j}+D_{j}$. $Q_{i}$ is used to represent the maximum buffer queue length of base station *i*.

Suppose that s[j][t][i] is used to represent the state of the packet *j* at time *t* on the base station *i*. If s[j][t][i]=1, this means that the packet *j* arrives (stays) on the base station *i* at time *t*; otherwise, s[j][t][i]=0. r[j] is used to represent the time when the packet *j* reaches the target node, that is, if s[j][r][t][i]=1, then i∈e. c[t][i] is used to represent the number of packets that the base station *i* waits to process in the cache queue at time *t*. In this paper, for the data packet *j*, the purpose of the transmission is to send it to the target node as soon as possible. Therefore, the objective function $f\left(j\right)$ is defined as follows:

(1) $$\begin{array}{c} f\left(j\right)=\frac{r\left[j\right]-A_{j}}{D_{j}} \end{array}$$

Obviously, $r\left[j\right]>A_{j}$. At the same time, in order to complete the scheduling before the deadline, we need to satisfy $r\left[j\right]<A_{j}+D_{j}$; therefore, 0<r[j]−Ajr[j]−AjDjDj≤1. That is, for any *j*, this satisfies $f\left(j\right)\in \left(0,\left.1\right]\right.$.

Therefore, we define the multi-sensor real-time routing scheduling problem for a given *I*, *J*, *T*, *A*, *e*, and nbr.

The multi-sensor real-time routing scheduling problem may contain many feasible solutions. To find an optimal solution among multiple feasible solutions, this paper takes the minimization of the objective function value in a feasible solution as the optimization objective. That is to say, for the scheduling results of *J* sensors, the largest $f\left(j\right)$ is selected as the representative of this set of solutions, and then the smallest of multiple feasible solutions is selected as the final problem solution. The mathematical model of the multi-sensor real-time routing scheduling problem S is as follows:

$$\begin{array}{c} \begin{array}{c} \min\left(\max_{j=0}^{J-1}f\left(j\right)\right)\\ s.t.\left\{\begin{array}{c} (1)\forall j\in \{0,1,\ldots ,J-1\},\\ r\left[j\right]\leq T-1\wedge r\left[j\right]-A_{j}\leq D_{j}\\ (2)\forall j\in \{0,1,\ldots ,J-1\},\\ s\left[j\right]\left[r\left[j\right]\right]\left[i_{e}\right]=1\wedge s\left[j\right]\left[A_{j}\right]\left[B_{j}\right]=1\\ \left(3\right)\forall j\in \left\{0,1,\ldots ,J-1\right\},i,i^{\prime }\in \left\{0,1,\ldots ,I-1\right\},t\in \left\{0,1,\ldots ,T-2\right\},\\ \mathrm{if}\;s\left[j\right]\left[t\right]\left[i\right]=s\left[j\right]\left[t+1\right]\left[i^{\prime }\right]=1,\;\mathrm{then}\;i^{\prime }\in nbr\left(i\right)\\ (4)\forall t\in \{0,1,\ldots ,T-1\},i\in \{0,1,\ldots ,I-1\},\\ c\left[t\right]\left[i\right]\leq Q_{i}\\ \left(5\right)\forall j\in \left\{0,1,\ldots ,J-1\right\},t\in \left\{0,1,\ldots ,T-1\right\},\\ \sum_{i=0}^{I-1}s\left[j\right]\left[t\right]\left[i\right]\leq 1 \end{array}\right. \end{array} \end{array}$$

The following explains the constraints of the problem S.

Constraints (1) and (2) show that, if there is r[j], it must be scheduled between the deadlines, and all scheduling beyond the deadline is not satisfied. If there is a packet that does not reach the endpoint, this condition is not satisfied. Therefore, as long as Constraints (1) and (2) are satisfied, all the data packets on the surface reach the destination node within the specified time.

Constraint Condition (3) indicates that data transmission can only be performed when two base stations are adjacent.

Constraint Condition (4) indicates that the cache of all base stations cannot overflow.

Constraint Condition (5) means that any sensor data can only be transmitted on one base station at any time.

**Figure 3:**
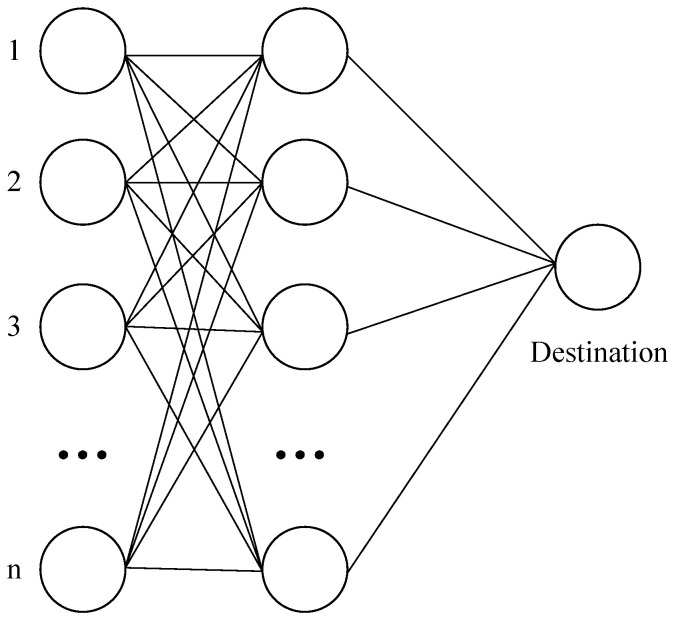
A special case of the multi-sensor real-time routing scheduling problem.

As shown in Figure 3, the network topology consists of *I* starting nodes, $I^{\prime }$ routing nodes, and a single terminal node *D*. For any i∈{0,1,…,I−1} and $i^{\prime }\in \left\{0,1,\ldots ,I^{\prime }-1\right\}$, let $i^{\prime }\in \mathrm{nbr}\left(i\right)$. Moreover, for any $i^{\prime }\in \left\{0,1,\ldots ,I^{\prime }-1\right\}$, it must hold that $D\in \mathrm{nbr}(i^{\prime })$. Let any $Q_{i}=\infty$. Clearly, the problem $S^{\prime }$ in Figure 3 is a subproblem of the problem S discussed in this paper. We now prove that $S^{\prime }$ is an NP-hard problem.

**Proof.**  Let W=I and $C=I^{\prime }$; this allows us to describe this problem as a multi-task, multi-processor scheduling problem [23] with *W* tasks and *C* processors. Nodes 1 to *n* in the figure are base stations with sensor data, and a column of nodes in the middle is a routable base station. Each sensor data can face the same n routing nodes after departure. It is evident that this transformation process has polynomial-time computational complexity. □

The problem proposed in [23] has been proven to be an NP-complete problem; therefore, the problem $S^{\prime }$ is NP-hard.

Since a simpler subproblem $S^{\prime }$ is NP-hard, the more complex problem *S* must also be NP-hard.

## 4. Algorithm Design

Since the NP-hard problem is difficult to solve effectively in polynomial time, this paper designs a variety of solving algorithms based on the ideas of the greedy algorithm, the shortest-path algorithm, and two heuristic algorithms and conducts experimental comparison and analysis to find different solutions to the problem under different conditions.

### 4.1. Algorithm Design Based on Greedy Strategy

Greedy strategy is a simple and rapid method to solve the problem. The characteristics of the greedy algorithm are step by step. The greedy algorithm does not consider the global optimal solution, but is only based on the current situation, and uses a certain measure to optimize the selection. Although the greedy algorithm cannot obtain the optimal solution in most cases, the greedy algorithm can obtain a ‘fairly good’ solution with a lower time complexity. For this problem, the most critical step is to select a fixed standard in each step of optimization selection. By selecting a fixed standard, it is necessary to be able to obtain a ‘not too bad’ solution at the end. The steps of using the greedy algorithm to solve this problem are as follows:

Step 1: Initialize the parameters, including the number of nodes *I*, the node set nbr(i), the export node $i_{e}$, the number of data packets *J*, the base station $B_{j}$ to which the data packet *j* belongs, the arrival time of the data packet *j*$A_{j}$, the deadline $D_{j}$, and the maximum buffer queue length of the base station $Q_{i}$;

Step 2: Sorting the data packets according to the absolute deadline in ascending order and selecting the first packet after sorting to start processing;

Step 3: If the current packet does not reach the export node $i_{e}$, save the current situation to the stack. Otherwise, skip to step 8;

Step 4: Select the base station that is adjacent to the current base station and has not been visited, and add it to the set to be selected. If the set to be selected is empty, the current base station is marked as visited, and the situation is recovered from the stack and jumped to step 2; if the set to be selected is not empty, the base station with the smallest load is selected as the next-hop base station;

Step 5: The arrival time is obtained based on the current load of the base station. If the buffer of the next-hop base station at the arrival time is full, the arrival time + 1, jump to step 5;

Step 6: Update c[T][I] and s[J][T][I] according to the base station number and arrival time of the next hop, and jump to step 3;

Step 7: Select a packet after the current packet; jump to step 3;

Step 8: Output results.

### 4.2. Design of Greedy-Based Genetic Algorithm

A genetic algorithm [24] is a heuristic algorithm. The sequence of each packet in the problem is encoded and can be solved by genetic algorithm. Since the results of the genetic algorithm are highly dependent on the initial solution, the results of the greedy algorithm are used as the initial solution in this paper. The specific steps are as follows:

Step 1: Use a greedy algorithm to solve the problem and use the sequence as the coding method. And retain the results of the greedy algorithm;

Step 2: Replicate the solution of a greedy algorithm as an individual of the population. Other individuals create an initial population by crossing c[T][I] in different packets;

Step 3: If the current round is greater than the number of iterations, jump to step 9;

Step 4: According to the fitness, randomly select the ‘number of individuals in the population’ for crossover;

Step 5: In the coding, the same site is put into the candidate set and randomly selected in the candidate set for crossover. Obtain offspring individuals. If it cannot be crossed, it is directly placed into the offspring individual;

Step 6: Coding mutation in the offspring set. According to the mutation rate, change the loci in the code. This site becomes randomly numbered as the base station connected to the adjacent last point;

Step 7: Merge the parent population with the offspring population and calculate the fitness;

Step 8: Generate the next group based on the fitness and roulette algorithm. Jump to step 3;

Step 9: Output results.

### 4.3. Design of Improved Genetic Algorithm Based on Dijkstra Coding

Dijkstra’s algorithmis an algorithm for finding the shortest path. This algorithm suits the shortest-path problem with a single source point on the graph G=〈V,E〉. During the algorithm’s operation, there will be a set of points S. The shortest-path values of all vertices and source points in the set S have been determined. The algorithm repeatedly selects the estimated minimum point u∈V−S. It adds this to S. This paper uses the Dijkstra algorithm to solve each packet along its shortest path to the endpoint and the calculation results.

In some cases, congestion will occur in the network if each packet passes the shortest path from the target node. In this case, the global delay is higher than that of some packets passing through non-shortest paths. Therefore, in the process of searching the shortest path in the Dijkstra algorithm, the non-shortest path should also be appropriately searched. These non-shortest paths should have the characteristics of ‘a cyclic’, and the shortest path difference is not too large.

Based on this idea, the Dijkstra algorithm is improved. In the search by the Dijkstra algorithm, the addition of non-minimum points is appropriately allowed in the process of repeatedly selecting the estimated minimum point. For example, the current distance is the shortest, and the worst case is not more than 5. This value can be adjusted according to the size of the graph. Through this improvement, the path derived from the Dijkstra algorithm can also appropriately contain non-minimum paths with little difference.

Genetic algorithms usually include genetic, mutation, hybridization, natural selection, and other processes. During the whole search process, it is always inclined to retain individuals with higher fitness. In the genetic algorithm, each needs to be encoded. In this problem, the individual is a specific case. In this case, each packet selects a path from the alternative set, and all packets select a feasible path. This constitutes a case of the individual of the problem. The case of each packet selecting a path constitutes the coding of the problem.

The Dijkstra algorithm can only maintain a single shortest path to a certain point, but only maintaining a single shortest path often falls into a local optimal solution, and it is difficult to find a better solution. For this problem, the key to improving the Dijkstra algorithm is to design rules for generating alternative paths. Considering that the greedy algorithm finds it difficult to use the shortest path in the early stage (or when the sample is small), the simple use of the Dijkstra algorithm will lead to multiple packets using the same path, which will lead to congestion. In this problem, the set of nodes that are touched in each step, and, in some cases, include the accessed nodes, is updated to the path set. Some of the additional alternative paths will also be updated with the Dijkstra algorithm update.

## 5. Experimental Analysis

### 5.1. Experimental Parameter Settings

Four different scale topologies are randomly generated to compare the algorithm’s advantages and disadvantages. Considering the real scene, the four topologies of different sizes are all non-fully connected graphs. The greedy algorithm, Dijkstra algorithm, genetic algorithm based on greedy algorithm, and genetic algorithm based on Dijkstra coding are compared and analyzed.

(1) Delay Performance Analysis

In order to compare different algorithms, the results obtained by different algorithms are compared in a ‘fitness’ manner. The higher the fitness, the better the solution and the smaller the delay. We compare the fitness of different algorithm scheduling results. After the same iteration, the greater the fitness of the task, the better the algorithm’s solution. The fitness calculation formula in this paper is

(2) $$\left(\tau_{Grd}-\tau_{*}\right)/\tau_{Grd}$$

where $\tau_{Grd}$ is greedy algorithm delay and $\tau_{*}$ are the delays of the algorithms to be evaluated.

(2) Convergence Speed Performance Analysis

If complete arrival cannot be achieved, the same round of iterations is performed on different algorithms to compare the proportion of completion. Under the same number of iterations, if the proportion of completion is higher, the algorithm is better. The calculation formula for the completion rate in this paper is the number of packets arriving before the deadline divided by the total number of packets.

Table 1 is the experimental parameter configuration of the analysis in this paper. Table 2 is the experimental parameter configuration of the convergence speed performance analysis in this paper.

In this experiment, fitness is the objective function of the problem, as shown in Equation (1). The value of fitness ranges from [0, 1]. Iters represents the number of iterations executed by the algorithm.

### 5.2. Experimental Result Analysis

In the experiment, the result of the greedy algorithm is used as its fitness benchmark (0) to minimize the impact of randomness on the experiment. In each experiment, the fixed graph and data packet input are run 10 times, and the average value of the fitness of the corresponding iteration times is taken as the result of the fitness of the 10 experiments.

**Figure 4:**
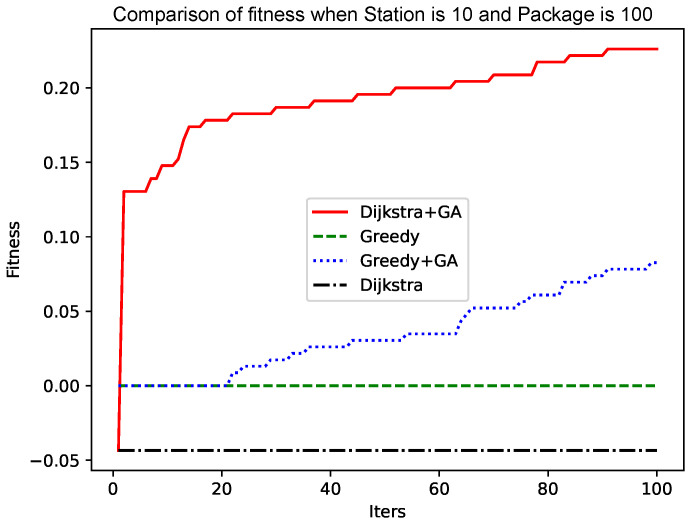
Comparison of fitness when Station is 10 and Package is 100.

When the number of points is 10 and the number of packets is 100, the experiment is carried out. The results are shown in Figure 4. Although the initial population using the Dijkstra algorithm is poor, the initial convergence is extremely fast, and a better solution can quickly be found. The convergence rate slows down in the later period, but it is constantly approaching the optimal solution. The faster initial convergence may be because the solution of the Dijkstra algorithm is poor. A better solution can be found after a simple crossover, mutation, and roulette algorithm. The genetic algorithm based on the greedy algorithm in the figure initially has a not-too-bad solution. Still, the initial crossover and mutation are not flexible, and to a certain extent, it is subject to the solution generated by the greedy algorithm. The medium term becomes faster because a better solution than the initial solution is generated, and the cross-matching roulette algorithm accelerates the convergence speed.

**Figure 5:**
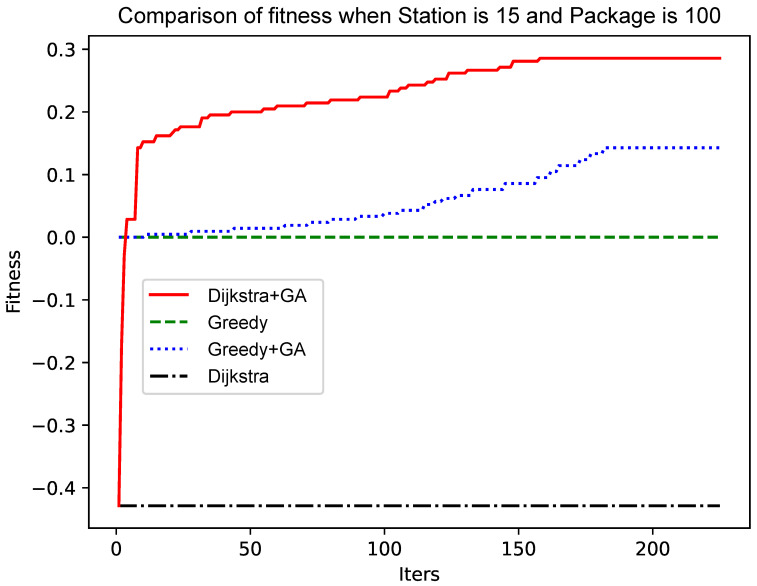
Comparison of fitness when Station is 15 and Package is 100.

**Figure 6:**
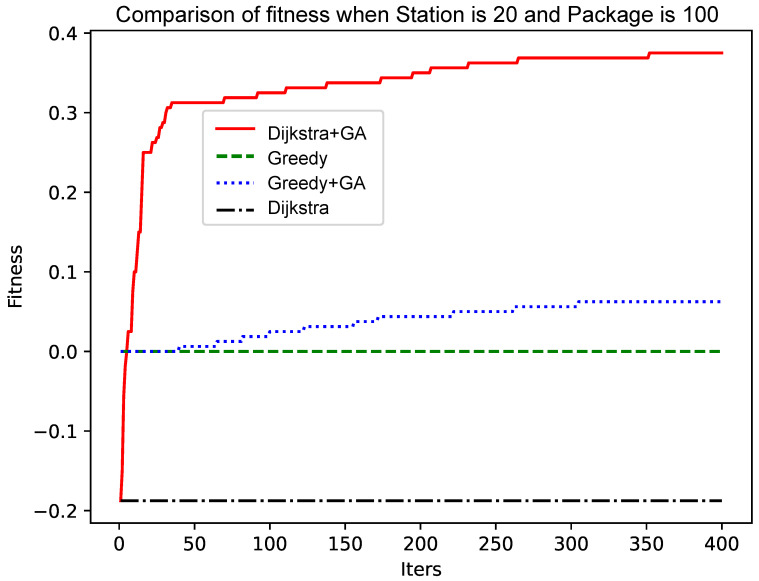
Comparison of fitness when Station is 20 and Package is 100.

As shown in Figure 5 and Figure 6, as the size of the graph increases, the genetic algorithm based on the greedy algorithm will slow the convergence speed. In contrast, the genetic algorithm based on the Dijkstra algorithm still has a fast convergence speed in the early stage, while the convergence speed in the later stage has no obvious change. The reason for this phenomenon is that, as the size of the graph increases, the ‘random’ variation will make it more and more difficult to find a better solution. The improved Dijkstra algorithm, although in the early shrinkage limit to understand the space, has a very high crossover and mutation efficiency. Therefore, as the size of the graph increases, the genetic algorithm based on the Dijkstra algorithm will be more advantageous.

**Figure 7:**
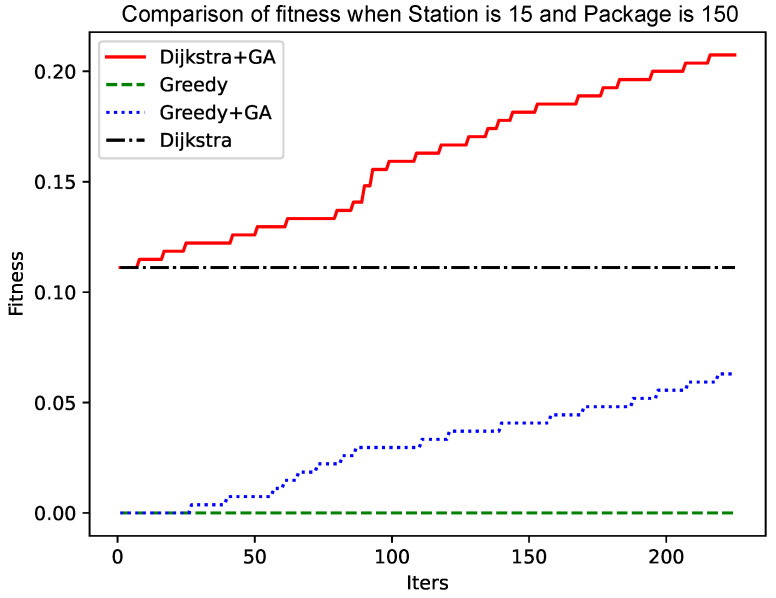
Comparison of fitness when Station is 15 and Package is 150.

**Figure 8:**
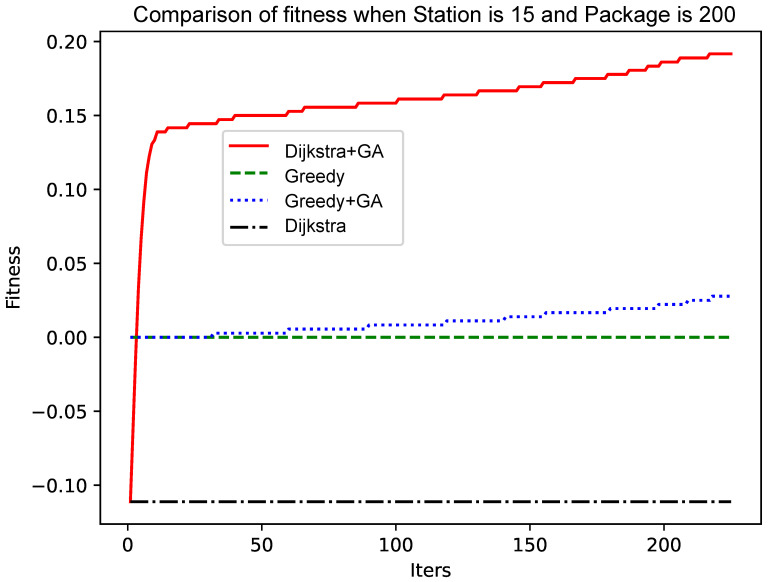
Comparison of fitness when Station is 15 and Package is 200.

**Figure 9:**
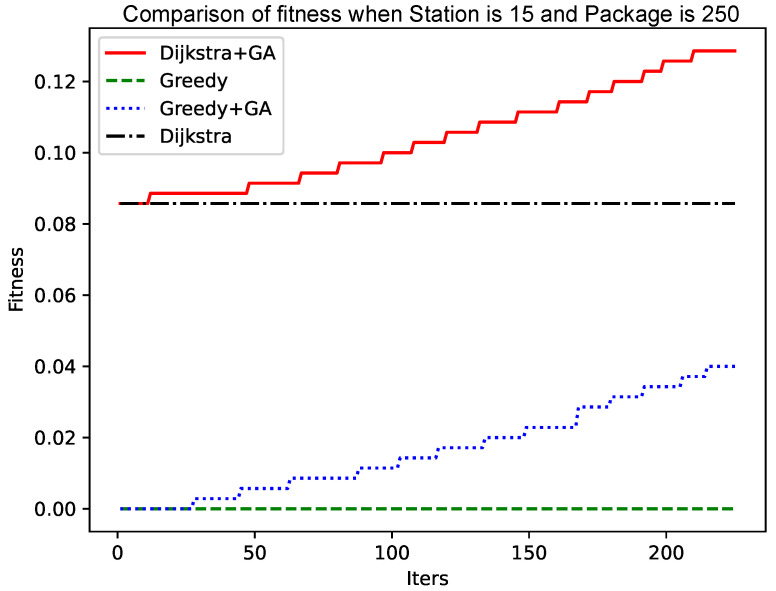
Comparison of fitness when Station is 15 and Package is 250.

As shown in Figure 4 and Figure 6, the number of data packets is increased, the genetic algorithm based on the greedy algorithm and the genetic algorithm based on the Dijkstra algorithm are compared, and the convergence speed does not change significantly. Comparing Figure 5 with Figure 7, Figure 8 and Figure 9, it is found that, when the Dijkstra algorithm can obtain a better solution, the convergence speed of the genetic algorithm based on the Dijkstra algorithm is relatively slow compared with the greedy algorithm. When the Dijkstra algorithm can obtain a better solution than the greedy algorithm, the genetic algorithm has a relatively fast convergence speed based on the greedy algorithm. The reason is that, when the initial solution is poor, there are more ways to iterate to a better solution; when the initial solution is poor, the method of iterating to the better solution is relatively few, and it is not easy to find.

**Figure 10:**
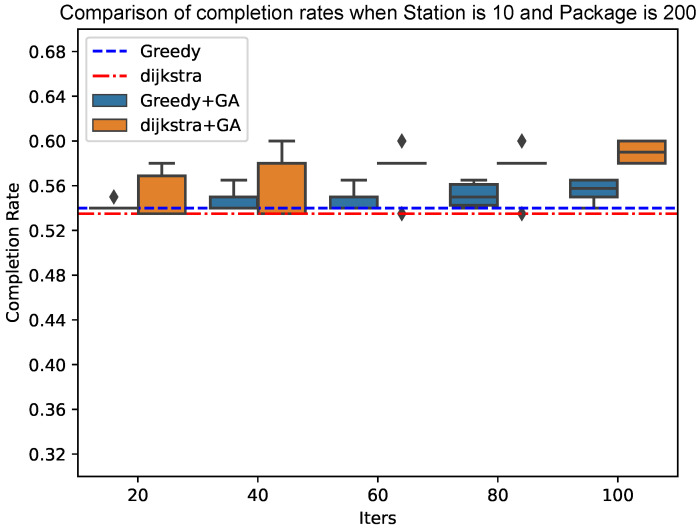
Comparison of completion rates when Station is 10 and Package is 200.

**Figure 11:**
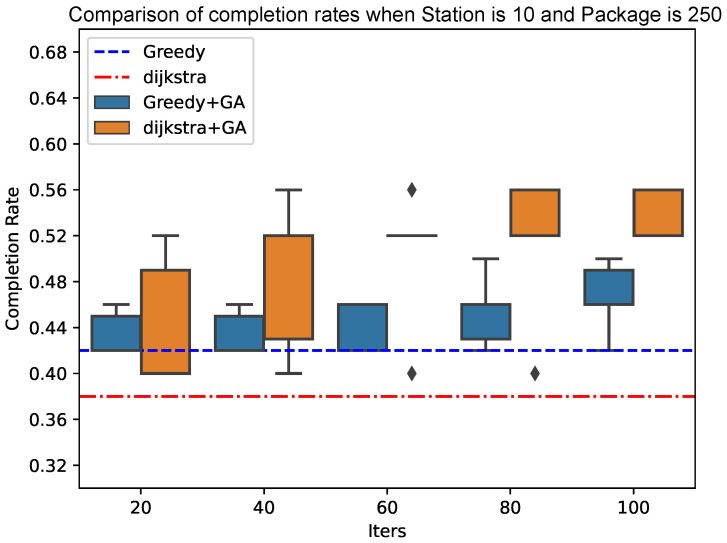
Comparison of completion rates when Station is 10 and Package is 250.

As shown in Figure 10 and Figure 11, the genetic algorithm based on Dijkstra has more advantages in the final result of the completion rate than the genetic algorithm based on the greedy algorithm. At the same time, the convergence speed of the genetic algorithm based on Dijkstra tends to be faster when the total number of packets increases; the advantages of the genetic algorithm based on Dijkstra will be more obvious.

In the experiments presented in this paper, the parameters of the genetic algorithm were set based on experimental data and optimized through multiple iterations of testing. After adjusting and selecting the optimal parameter combination, the final settings were chosen. In practical engineering applications, the parameter settings of the genetic algorithm can significantly impact the results. Typically, optimization techniques such as particle swarm optimization are used for parameter tuning. As there is already related work on this topic, we do not elaborate further on this aspect in this paper.

## 6. Conclusions

This paper studied the real-time data transmission problem of the IoT for underground mining safety. The current work was analyzed, and it was demonstrated that existing approaches cannot effectively solve the real-time data transmission problem of IoT in underground mines. Addressing the limitations of current approaches, this paper analyzed the tunnel structure of IoT in mines and proposed a hybrid network topology that combines wireless and wired transmission. A multi-sensor, multi-base-station real-time routing scheduling problem was defined, and it was proven to be NP-hard. Three different algorithms were proposed, and experiments were designed to analyze the delay performance of these algorithms under varying node sizes and packet numbers. At the same time, the computational complexity of the three algorithms was analyzed by evaluating their convergence speeds. The method proposed in this paper can be applied to the real-time routing scheduling of multi-sensors in IoT systems in mining environments and provides a feasible solution with relatively better constraints before the deadline.

In practical applications, due to the complex structure of mines, the reliability of wireless signals is difficult to ensure. Additionally, since the algorithm proposed in this paper requires good clock synchronization between base stations, but devices in underground mines find it challenging to maintain synchronization through low-performance crystal oscillators, these issues must be addressed. In practical applications, a polling method should be used to ensure clock synchronization, and algorithms like CSMA should be appropriately considered to ensure the reliability of data transmission from sensors to base stations.

The main direction for future research is to improve the execution efficiency of the algorithm and analyze the optimization algorithm design approach for specific scenarios, such as dynamic programming. The goal is to propose an optimized scheduling algorithm with pseudo-polynomial time complexity.

## Figures and Tables

**Table 1:** Delay performance analysis parameter configuration.

Network Topological Parameter	Task Parameter	Genetic Algorithm Parameters Based on Greedy Algorithm	Genetic Algorithm Based on Dijkstra Encoding
Station: 10	Package: 200	Mutation: 0.8	Mutation: 0.8
Station: 10	Deadline: 45	Population: 100	Population: 100
Station: 15	Package: 100	Mutation: 0.8	Mutation: 0.8
Cache: 30	Deadline: 35	Population: 100	Population: 100
Station: 15	Package: 150	Mutation: 0.8	Mutation: 0.8
Cache: 30	Deadline: 40	Population: 100	Population: 100
Station: 15	Package: 200	Mutation: 0.8	Mutation: 0.8
Cache: 30	Deadline: 45	Population: 100	Population: 100
Station: 15	Package: 250	Mutation: 0.8	Mutation: 0.8
Cache: 30	Deadline: 50	Population: 100	Population: 100
Station: 20	Package: 100	Mutation: 0.8	Mutation: 0.8
Cache: 40	Deadline: 45	Population: 100	Population: 100

**Table 2:** Convergence speed performance analysis parameter configuration.

Network Topological Parameter	Task Parameter	Genetic Algorithm Parameters Based on Greedy Algorithm	Genetic Algorithm Based on Dijkstra Encoding
Station: 10	Package: 200	Mutation: 0.8	Mutation: 0.8
Cache: 30	Deadline: 45	Population: 100	Population: 100
Station: 10	Package: 250	Mutation: 0.8	Mutation: 0.8
Cache: 30	Deadline: 45	Population: 100	Population: 100

## Data Availability

All experimental data are presented in the paper.
